# An automatic diagnosis model of otitis media with high accuracy rate using transfer learning

**DOI:** 10.3389/fmolb.2023.1250596

**Published:** 2024-03-21

**Authors:** Fangyu Qi, Zhiyu You, Jiayang Guo, Yongjun Hong, Xiaolong Wu, Dongdong Zhang, Qiyuan Li, Chengfu Cai

**Affiliations:** ^1^ Department of Anesthesiology, The Seventh Affiliated Hospital, Sun Yat-sen University, Shenzhen, China; ^2^ School of Medicine, Xiamen University, Xiamen, China; ^3^ National Institute for Data Science in Health and Medicine, Xiamen University, Xiamen, China; ^4^ Zhongshan Hospital Affiliated to Xiamen University, Xiamen, China; ^5^ College of Otorhinolaryngology Head and Neck Surgery, Xiamen Haicang Hospital, Xiamen, China

**Keywords:** chronic suppurative otitis media (CSOM), middle ear cholesteatoma, CT images, computer-aided diagnosis (CAD), transfer learning (TL)

## Abstract

**Introduction:** Chronic Suppurative Otitis Media (CSOM) and Middle Ear Cholesteatoma are two common chronic otitis media diseases that often cause confusion among physicians due to their similar location and shape in clinical CT images of the internal auditory canal. In this study, we utilized the transfer learning method combined with CT scans of the internal auditory canal to achieve accurate lesion segmentation and automatic diagnosis for patients with CSOM and middle ear cholesteatoma.

**Methods:** We collected 1019 CT scan images and utilized the nnUnet skeleton model along with coarse grained focal segmentation labeling to pre-train on the above CT images for focal segmentation. We then fine-tuned the pre-training model for the downstream three-classification diagnosis task.

**Results:** Our proposed algorithm model achieved a classification accuracy of 92.33% for CSOM and middle ear cholesteatoma, which is approximately 5% higher than the benchmark model. Moreover, our upstream segmentation task training resulted in a mean Intersection of Union (mIoU) of 0.569.

**Discussion:** Our results demonstrate that using coarse-grained contour boundary labeling can significantly enhance the accuracy of downstream classification tasks. The combination of deep learning and automatic diagnosis of CSOM and internal auditory canal CT images of middle ear cholesteatoma exhibits high sensitivity and specificity.

## Introduction

Otitis media is a prevalent ear disease that affects a significant portion of the global population, with an estimated 65 to 350 million individuals affected worldwide (World Health Organization, 2004). In developing countries, the prevalence of Chronic Suppurative Otitis Media (CSOM) ranges from 0.4% to 33.3% (Kaur et al., 2017). Our study mainly focuses on non-invasive temporal bone CT images, in order to help clinicians quickly get a relatively accurate preliminary diagnosis and lay the foundation for further judgment of whether patients need surgical treatment.

Otitis media is classified into three categories: acute otitis media, chronic otitis media (COM), and middle ear cholesteatoma. Chronic otitis media typically exhibits more pronounced pathological changes on CT images due to its protracted course, whereas acute otitis media does not usually display this characteristic. Consequently, it is often recommended that patients with chronic otitis media undergo internal auditory canal CT scan to assess their condition. Chronic otitis media is further divided into two subcategories: chronic non-suppurative otitis media and chronic suppurative otitis media (CSOM) (Schilder et al., 2017). CSOM and Middle Ear Cholesteatoma are two typical otitis media diseases that are diagnosed primarily through temporal bone CT scans (Fukudome et al., 2013; Lustig et al., 2018). CSOM typically occurs following improper treatment of acute otitis media, often resulting in tympanic membrane perforation and persistent middle ear purulence (Ahmad et al., 2022). Middle ear cholesteatoma, on the other hand, is the pathological outcome of abnormal accumulation of keratin squamous epithelium, primarily composed of keratinized, exfoliated epithelium. It often accumulates in the middle ear, with a tendency to erode the ossicular chain, tympanic wall, and/or mastoid area (Sun et al., 2011; Jang et al., 2014)^.^

Clinicians usually identify CSOM and middle ear cholesteatoma through CT scanning of the internal auditory canal. However, CT reports of these two types both show erosion and/or loss of the ossicular chain with diffuse abnormal soft tissue shadow (Madabhushi and Lee, 2016). Theoretically, the two types differ in bone erosion margins and soft tissue shadow contours: The soft tissue shadow of cholesteatoma has a smooth, clear outline, while that of CSOM lacks a clear outline and is often accompanied by pus accumulation. In addition, the edge of the bone erosion caused by CSOM is serrated, while bone destruction caused by cholesteatoma is frequently surrounded by a ring of sclerosis. Therefore, our group proposed using deep learning to differentiate between CSOM and cholesteatoma to achieve more accurate clinical diagnoses based on the theoretical differences between these two diseases (Kemppainen et al., 1999; Yorgancılar et al., 2013).

Deep learning techniques have seen widespread use in the medical field in recent years, enabling the extraction of key features from patients to facilitate predictive modeling (Elfiky et al., 2018). Transfer learning is a deep learning technique that involves leveraging knowledge gained from solving one problem to address another related problem. It is particularly useful when the amount of labeled data for the target task is limited. By leveraging transfer learning, a pre-trained model developed for one task can be fine-tuned and adapted for another task with different data but similar features. This approach enables the model to benefit from the knowledge learned by the pre-trained model on a larger dataset and adapt it to the new task by making only minor adjustments to the model’s architecture or parameters. A bunch of researches have uncovered that superiority of transfer learning over traditional strategy. S. Deepak implement brain tumor classification using deep CNN features via transfer learning (Deepak and Ameer, 2019). For tuberculosis detection, a VGGNet based model had been proposed combining transfer learning (Ahsan et al., 2019). Dube S presented an automatic content-based image retrieval system for brain tumors on contrast-enhanced MRI (Dube et al., 2006).

Given the small morphological differences between various types of otitis media, the challenge of manual identification, and the unclear contour of lesions, we sought to establish a transfer learning framework by integrating coarse-grained labeled contour information as pre-trained data and employing the CNN model skeleton to extract high-level features from internal auditory canal CT images. Specifically, the deep representation of images obtained through pre-training was utilized to accurately classify CSOM, middle ear cholesteatoma, and normal samples in downstream tasks.

In conclusion, this paper’s key contributions are:

1. We propose a transfer learning-based framework that utilizes coarsely annotated segmentation data as input for pretraining the model. The proposed model effectively extracts implicit information from the data and can subsequently be used for classification prediction.
2. We propose an end-to-end learning model that can effectively improve the accuracy of middle ear infection classification prediction. Our proposed model outperforms non-pretrained models in all metrics.
3. In the field of deep learning combined with medicine, we look forward to replacing the heavy and repetitive manual labeling task with more mature machine automated labeling.
4. Our combination of otological diseases and computer learning can increase the coverage of related research and provide more precise and diversified help for clinicians in diagnosis and treatment.

## Materials and methods

### Data acquisition

We conducted a retrospective study at Zhongshan Hospital Affiliated to Xiamen University to investigate patients diagnosed with otitis media and middle ear cholesteatoma from 2012 to 2021. This study was approved by the ethics committee and informed consent was waived due to the retrospective nature of the study. The inclusion criteria for the study were based on pathology or medical history, ear examination, audiogram, and imaging examination of the surgical side of the ear. We referred to the previous medical records of the hospital to obtain specific diagnosis results. A total of 6,967 axial high-resolution CT images of temporal bone were collected from 180 patients, including 27 female patients with middle ear cholesteatoma, 31 male patients with middle ear cholesteatoma; 50 female patients and 51 male patients with chronic otitis media, including 40 females and 39 males with CSOM, and Chronic otitis media with effusion, including 10 females and 12 males. Besides, there were 15 normal controls (6 females and 9 males), and 2 children with middle ear cholesteatoma, 2 children with CSOM (less than 10 years old), and 2 normal controls. Except for children, the patients collected were between 30 and 80 years of age. All the patients information has been shown in Table 1.

**TABLE 1:** The collected patients information.

	Female (30–80 years old)		Male (30–80 years old)	Pediatric patients (<10 years old)
Middle Ear Cholesteatoma	**27**		**31**	**2**
CSOM	**40**		**39**	**2**
Chronic Otitis Media with Effusion	**10**		**12**	**0**
Normal	**6**		**9**	**2**
Totall	**83**		**91**	**6**

Data sources: Zhongshan Hospital Affiliated to Xiamen University.

Note: numbers represent the quantity of patients.

The CT features of chronic otitis media with effusion (COME) are often very similar to those of chronic suppurative otitis media (CSOM), with both conditions typically presenting with varying degrees of effusion in mastoid cells. As such, we excluded a total of 412 axial CT data from 22 patients diagnosed with COME. For each patient, we selected approximately 10–20 CT images that showed well-defined lesions. Ultimately, we used 410 CSOM CT images, 398 middle ear cholesteatoma CT images, and 211 normal CT control images for our analysis.

## CT scanner settings

Temporal bone CT is derived from GE LightSpeed 64-row volume CT. Its detector is the core technology of multi-slice spiral CT. The detector arrangement adopts 64 × 0.625 mm detector unit to ensure the maximum coverage of 40 mm/circle at present, and at the same time, it can also perform sub-millimeter thick scanning in any mode. The isotropic resolution is up to 0.30 mm, which ensures a large range of volume acquisition and high resolution acquisition. The CT imaging parameters used were as follows: CT collimator 128 × 1.0 mm, field of view 220 × 220 mm, matrix size 1,024 × 1,024, voltage 120 kV, current 240 mAs, and axial CT slice number 30–50 per scan.

### Data marking

**FIGURE 1:**
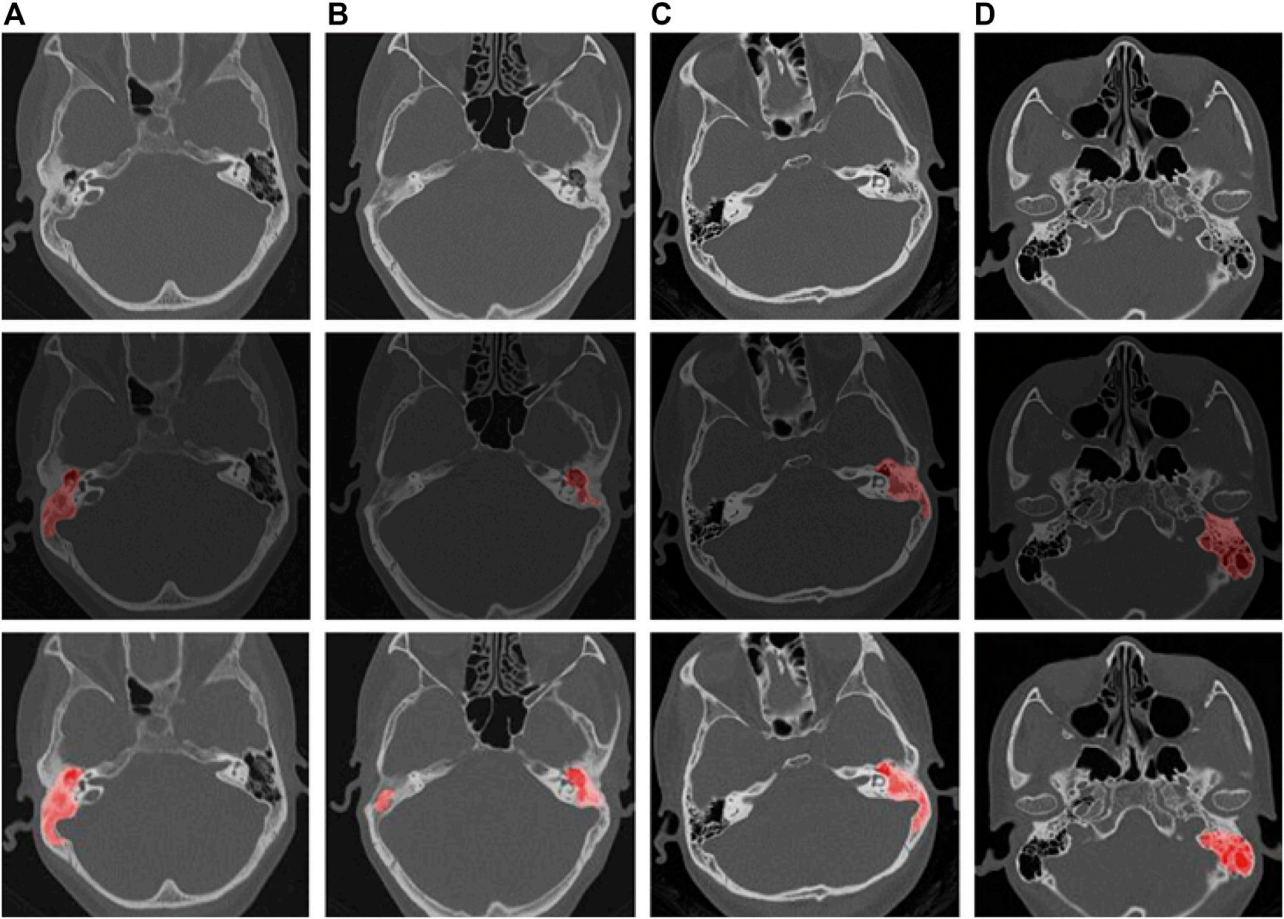
Schematic representation of labeling and pre-trained model predictions for middle ear cholesteatoma and CSOM. (**(A, B)** are cholesteatomas, and **(C, D)** are CSOM).

The CT findings of chronic suppurative otitis media are often difficult to distinguish from middle ear cholesteatoma. To accurately identify middle ear cholesteatomas, we marked local or isolated cholesteatomas in the erosion area of the incudostapedial joint or hammer-incus joint in the rotation plane of the middle tip of the cochlea. We also highlighted areas of bone destruction within the tympanic sinus, epitympanic region, or mastoid process in other levels. Additionally, we marked any irregular soft tissue shadows with smooth edges on any plane. In contrast, when marking CT images of suppurative otitis media, we identified the soft tissue shadow around the auricle in the tympanic cavity, the sclerotic hyperplasia part of the mastoid, and the bone with uniform density and serrated edge in the tympanic sinus at the vestibular level. These characteristics helped distinguish them from the sclerosing ring that is formed by the compression of a cholesteatoma. At the apical spiral layer of the cochlea, we marked the hammer-incus joint of the ossified epitympanic, the “ice cream cone-like structure,” and the serrated bone around the mastoid cavity and sinus. At the bottom spiral level of the cochlea, we marked the thickened mucosa on the promontory surface. Finally, at the mastoid level, we marked the erosion of the mastoid bone and the thickening of the mucous membrane caused by suppurative effusion (Gomaa et al., 2013; Zelikovich, 2004). We provide visual examples of typical lesion markers and predictions for these two diseases in Figure 1.

The original data was stored in the Dicom format, which we converted into PNG image data using MicroDicom software. This step allowed us to separate the patient’s personal information from the image, thereby ensuring patient privacy.

To mark the lesion area on each image, we enlisted the help of a team consisting of five professional otolaryngologists and two radiologists. They used polygonal markers on LabelMe software to eliminate background interference and generate unified coordinates for each lesion area.

### Data pre-processing

To improve the robustness of our model, we applied various image data augmentation and processing techniques during the training process. Specifically, we randomly transformed the input images by performing horizontal and vertical translations, flipping, rotation, slight scaling, and adjustments to hue, contrast, and numerical values.

In order to balance the size of our training model and the time required for training, we scaled all images to a uniform size of 224 × 224 using bilinear interpolation. This allowed us to efficiently process and train on a large dataset of images while still maintaining a high level of accuracy and performance in our final model.

### Model architecture and training strategy

We utilized the nnUnet (Isensee et al., 2021)architecture as the foundation for our deep learning model to extract critical features from CT images. This model has demonstrated exceptional performance in various medical image segmentation tasks. Our model consists of two branches: coarse-grained segmentation task and exact classification task.

In our workflow, we first pre-trained a model for lesion segmentation, which includes the nnUnet skeleton and a pixel-level prediction head that outputs three classification results for each pixel: CSOM, middle ear cholesteatoma, or normal samples. On the back of the above-mentioned process, our goal was to acquire a well-trained backbone that could extract underlying information containing pixel-level features, which would then be fine-tuned for picture-level classification. We trained this model using the gradient descent algorithm until convergence was achieved.

The nnUNet model is composed of an encoder and a decoder. The encoder reduces the image size layer by layer while capturing features of varying granularity from different images. It consists of seven layers, each containing {1, 3, 4, 6, 6, 6, 6} blocks, with each block containing two convolutional layers, two activation layers, and two normalization layers. Successive layers are directly connected with a pooling layer, which reduces the image size by half. The first layer of the encoder contains 32 features, and the number of features in each subsequent layer doubles but does not exceed the maximum number of features, which is 512.

The decoder has six layers, each consisting of {2, 2, 2, 2, 2, 2} blocks. These layers use linear interpolation upsampling to increase the image size. The encoder and decoder are connected using residual layer hopping. The decoder outputs the hidden variables of the image as inputs to both the pixel-level projection head and the image-level projection head for further processing.

Once the model was successfully trained, we extracted the hidden variables before the pixel-level prediction head of the image as inputs for the downstream classification prediction head. This allowed us to efficiently classify images with high accuracy by leveraging the previously extracted features.

**FIGURE 2:**
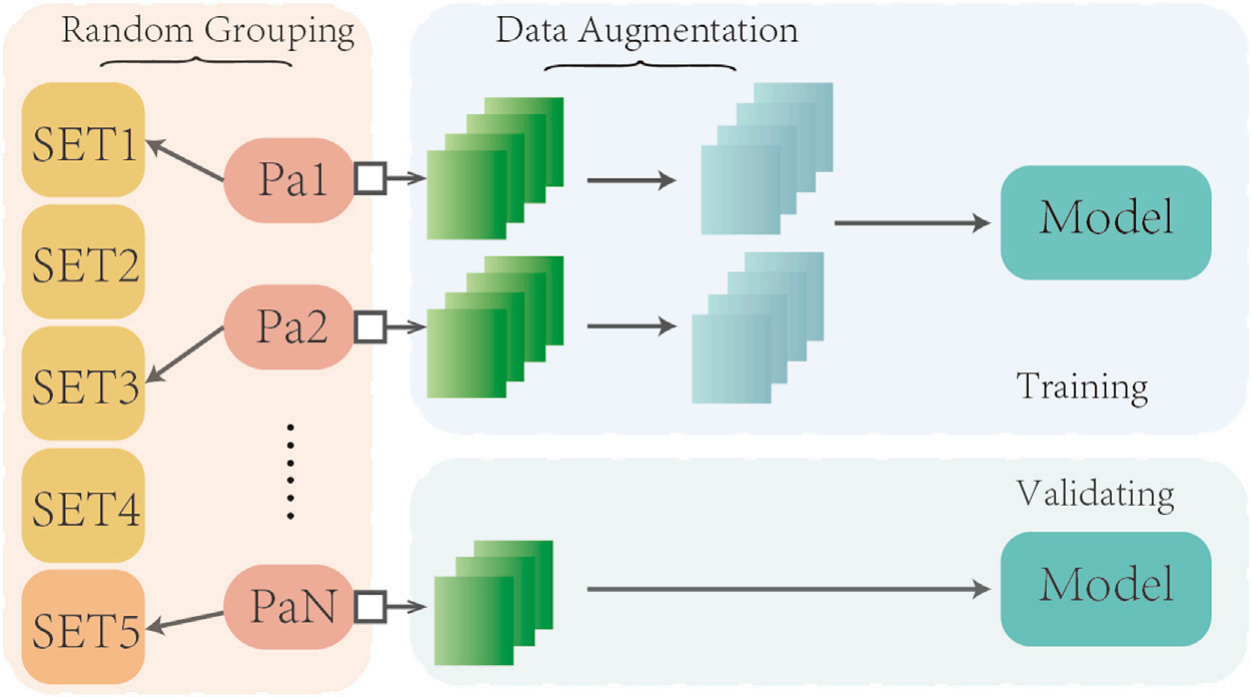
Schematic diagram of five-fold cross validation.

We employed a five-fold cross-validation approach to train and evaluate our model (see Figure 2). Given that a series of adjacent CT images from the same subject tend to exhibit strong similarities, we took care to avoid overfitting due to data leakage. Specifically, during the training process, we randomly divided the dataset into a training set and validation set at a ratio of 4:1, based on the subject’s name. This ensured that CT images from the same subject were assigned to either the training or validation set, but not both.

**FIGURE 3:**
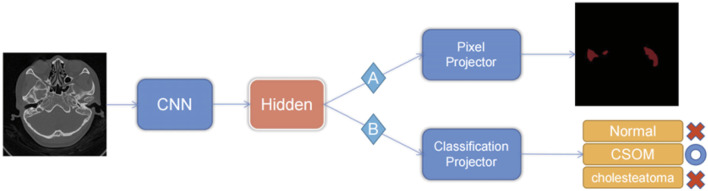
Transfer learning strategy.

Our models were compiled using Python 3.8, trained with PyTorch version 1.10, and accelerated with Nvidia A100 high-performance GPUs. During the training process, we set the maximum training epoch to 500 epochs, with a training batch size of 8 samples. We used Adam as the model optimizer, with an initial learning rate of 0.001. The dynamic learning rate decreased gradually with each increase in training batch until it reached 10e-5. For pre-training optimization, we utilized the cross-entropy of each pixel classification of the image. Downstream training utilized the cross-entropy of the image classification as the loss function. Our specific workflow is depicted in Figure 3 for further visualization.

(The pre-training phase uses the pixel-level labels of route A to train the CNN, and the pixel predictor is responsible for output the category of each pixel. When performing the downstream picture classification task, according to route B, the pre-trained model is used to fine-tune the neural network through the category classifier, and the final picture prediction result is output.)

## Result

In the upstream segmentation task, our deep learning model achieved a mean Intersection of Union (mIoU) index of 0.5376, indicating excellent performance in accurately removing background noise. Subsequently, we employed this well-performing model for the downstream fine-tuning step, where we aimed to classify otitis media into three distinct categories. Our model achieved a micro-f1 index of 92.33%, a significant improvement of 4.83% compared to the benchmark model.

**FIGURE 4:**
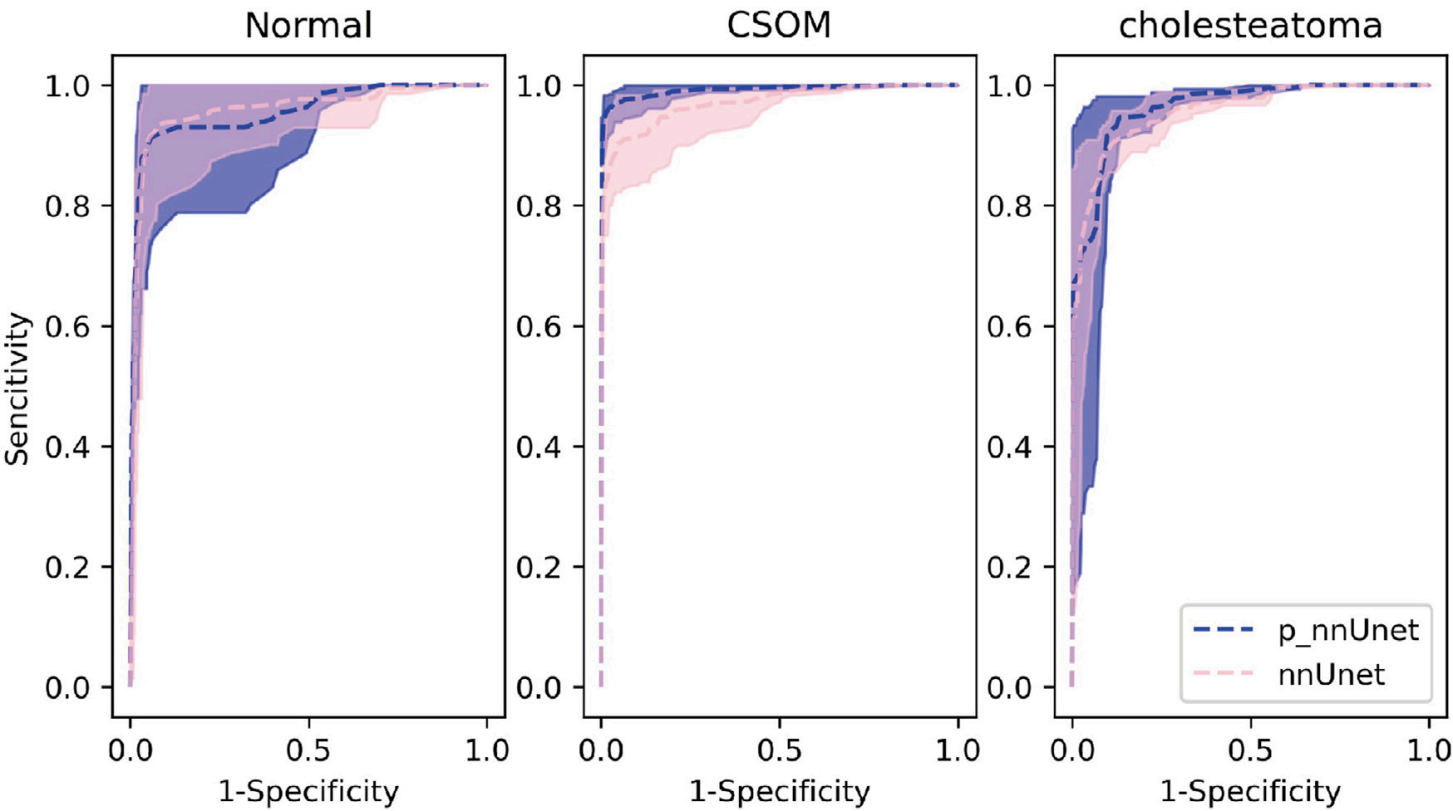
Receiver curves for each category of the model.

On the other hand, the pre-trained model exhibits an overall area under the receiver curve of 0.9689, which is slightly higher than that of the benchmark model which reach 0.9603. As is depicted in Figure 4, it can be observed that the performance in distinguishing chronic suppurative otitis media (CSOM) is the best, by a margin of 8.15%, as is showed in Table 2. These results indicate that the pre-trained model has a superior ability to accurately classify CSOM cases compared to the benchmark model. These results highlight the potential of deep learning technology in medical image analysis and its ability to significantly improve diagnostic accuracy and treatment outcomes.

$$m\text{IoU}=\frac{1}{\left|D\right|}\sum_{i\in D}\frac{{\text{TP}}_{i}}{{\text{TN}}_{i}+{\text{FP}}_{i}+{\text{FP}}_{i}}$$

**TABLE 2:** Comparison of results.

		Accuracy	Auc
nnUnet	Normal vs. the others	0.8732 ± 0.1694	0.9558 ± 0.0454
	CSOM vs. the others	0.8619 ± 0.0776	0.9695 ± 0.0212
	Cholesteatoma vs. the others	0.9013 ± 0.0394	0.9555 ± 0.0242
p_nnUnet	Normal vs. the others	0.8873 ± 0.1593	0.9530 ± 0.0526
	CSOM vs. the others	0.9434 ± 0.0343	0.9916 ± 0.0069
	Cholesteatoma vs. the others	0.9290 ± 0.0170	0.9622 ± 0.0316

p represents the fine-tuning results after using the pre-trained model. The above results are the means after five-fold cross-validation. The mIoU index is used to describe the average ratio of intersection and union of all pixel categories in the image segmentation task. In this experiment, the background normal tissue categories were removed to obtain more accurate prediction results. mIoU is described as follows.

Note: In the above equation, FN is the false negative class, FP is the false positive class, FP is the true class, and D represents the CSOM and cholesteatoma set.

On the other hand, we demonstrate the disparity in accuracy between AI models and manual diagnosis by clinicians. We intentionally randomized and combined a total of 1013 CT images of middle ear cholesteatoma, CSOM, and normal control images, while effectively concealing the actual diagnostic labels. These images were then distributed to both the manual diagnosis group and the model diagnosis group for a double-blind evaluation of diagnostic accuracy. The comprehensive test results are shown in Tables 3, 4, and it can be observed that CSOM and cholesteatoma exhibit a high misdiagnosis rate.

**TABLE 3:** Diagnostic accuracy results of manual diagnostic group.

The clinical experts (*n* = 3)/Real diagnose (n = 3,039)	COSM (*n* = 1,182)	Cholesteatoma (*n* = 1,224)	Normal (*n* = 633)
CSOM	948 (80.20%)	489 (39.95%)	48 (7.58%)
Cholesteatoma	222 (18.78%)	708 (57.84%)	9 (1.42%)
Normal	12 (1.01%)	27 (2.20%)	576 (90.99%)

The horizontal represents the actual diagnosis results, while the vertical represents the manual diagnosis results.

**TABLE 4:** Diagnostic accuracy results of model diagnosis group.

The AI model/Real diagnose (*n* = 1,013)	COSM (*n* = 394)	Cholesteatoma (*n* = 408)	Normal (*n* = 211)
CSOM	336 (85.28%)	16 (3.92%)	7 (9.52%)
Cholesteatoma	44 (11.17%)	336 (82.35%)	2 (0.95%)
Normal	14 (3.55%)	20 (4.90%)	188 (89.52%)

The horizontal represents the actual diagnosis results, while the vertical depicts the diagnostic outcomes of the deep learning model.

Our experimental results show that in the otitis media classification task, the use of contour boundary labeling can well improve the accuracy of downstream classification tasks, and the area under the receiver operating characteristic curve is better than that of the non-pre-trained model shown in Figure 4. These results indicate that the predictive power of our model on this task has the possibility of real-world application.

(p-represents the fine-tuning results after using the pre-trained model. In the multi-classification ROC curve, the positive samples belong to a particular category while the negative samples belong to all other categories combined. Based on this distinction, the true positive rate and false positive rate have been accurately calculated).

## Discussion

Otitis media is characterized by a prolonged course of illness, high incidence, easy recurrence, conductive deafness, and potentially fatal intracranial infection (Otten and Grote, 1990; Hutz et al., 2018). A case analysis conducted in a public hospital in the United States revealed that the incidence of postoperative complications associated with complex chronic otitis media with middle ear cholesteatoma was similar to that observed in developing regions (Greenberg and Manolidis, 2001). As a result, early diagnosis, intervention measures, and clinical management of this disease are especially crucial, regardless of whether one resides in developed or developing regions. In our study, we utilized CT images of CSOM and middle ear cholesteatoma labeled by medical experts as the training set for our algorithmic model. Our algorithm model accurately predicted unlabeled CT images with a high degree of precision, achieving excellent agreement between predicted lesion types and actual clinical findings.

So far, CT scan and Endoscopy of ear, as the classical methods for the diagnosis of various types of otitis media, are still the latest diagnostic methods (Gomaa et al., 2013; Zelikovich, 2004). The golden standard for the diagnosis of CSOM and middle ear cholesteatoma is intraoperative histopathological examination. However, it takes a long time to make a preliminary diagnosis of one single patient. Despite efforts to reduce the prevalence of Chronic Suppurative Otitis Media (CSOM) in underdeveloped areas, clinical diagnosis, treatment, and prognosis of the disease remain suboptimal. Recent epidemiological investigations have shown that CSOM has shifted to a population dominated by adults, despite a decrease in overall prevalence during the past 2 decades (Orji et al., 2016). Additionally, an Australian survey highlighted the high incidence of CSOM and middle ear cholesteatoma among impoverished individuals and the need for early diagnosis (Benson and Mwanri, 2012).

Deep learning has emerged as a valuable tool in various medical fields. A substantial amount of research on deep learning applied to clinical datasets, using high-quality medical examination images, has showcased its efficacy in defining patient categories, identifying and locating lesions, and other relevant tasks (Wang et al., 2020). With our transfer learning model, medical researchers can avoid the time-consuming and resource-intensive process of training models from scratch, while also benefiting from the wealth of knowledge captured in existing non-medical datasets. In the field of computational vision, pre-trained models have become a commonly used tool in many applications, particularly in addressing medical imaging challenges. These challenges can arise from imaging modalities such as X-ray, Magnetic Resonance Imaging (MRI), CT scan, and Ultrasound data. Many works have demonstrated the potential of pre-trained models to improve diagnostic accuracy, reduce processing time, and assist in the development of automated diagnosis systems. Our transfer learning model could also be used in other diseases which need CT scan or endoscope or any examinations that take images as the method to diagnose. Once the medical examination images are too similar to find the differences, our model could give several suggestions in differential diagnosis based on the previous history image labels.

Among the different types of otitis media, there are varying methods for diagnosis and treatment. However, the CT image features tend to be similar across these types, which can pose challenges for clinicians in terms of differential diagnosis. Such challenges can lead to delays in proper treatment, and potentially result in errors or overmedication. Moreover, the COVID-19 patients were found to have relationship with Otitis media (Choi et al., 2022), they demand to be diagnosed earlier than before, as otitis media always intend to recurrence and even cause Sensorineural-hearing-loss (Xia et al., 2022). As a result, achieving rapid differential diagnosis for otitis media is crucial to ensure optimal patient outcomes, in this situation, an efficient diagnosis can be given using our transfer learning model.

However, our transfer learning models have shown some limitations in classifying certain ear diseases. For instance, when differentiating between secretory otitis media and suppurative otitis media, deep learning models tend to confuse the two because their CT scans are very similar. This could be attributed to inadequate data sets. As such, there is a need for more comprehensive and diverse medical data to improve the accuracy of diagnostic models used to differentiate between various ear diseases. Moreover, our model showed significant differences in lesion information extraction. For instance, some predicted lesions would perform fewer or more lesions compared to those marked by medical experts. Also, in images with unclear lesions, there were discrepancies in identifying the lesion. For example, the images of the mastoid layer of chronic suppurative otitis media often have varying degrees of mucosal thickening due to chronic inflammation, while the images of the mastoid layer of chronic secretory otitis media show fluid levels caused by chronic effusion. These conditions are quite similar, with only slight differences in the contour of the mucosal within the mastoid bone. In most cases, our transfer learning networks could detect and label prominent lesions such as large soft tissue shadow of middle ear cholesteatoma, eroded bone structure surrounded by soft tissue shadow, and eroded bone structure of chronic suppurative otitis media. Unfortunately, the CT images of chronic otitis media with effusion (COME) do not have the typical erosive features of CSOM and middle ear cholesteatoma. Therefore, our research group excluded the CT images of chronic secretory otitis media and focused solely on collecting CT images of middle ear cholesteatoma and chronic suppurative otitis media as the objects of our study. In the future, when the amount of data collection is large enough, we will continue to promote the application of new migration models to this type of classification project.

What’s more, due to the limited clinical applications of deep learning and the laborious, time-consuming nature of acquiring supervised data such as lesion regions, our research group aims to identify alternative weakly supervised signals for model transfer learning pre-training. “Human-in-the-loop” is an effective interactive mode between doctors and models, which can provide weakly supervised signals and ensure continuous learning of the model. This approach also represents a practical scenario for the clinical application of the model. This can help reduce manual labeling costs and improve overall prediction performance. In the future, we hope to replace the repetitive, cumbersome task of manual labeling with more advanced machine automated labeling techniques in the field of deep learning combined with medicine.

Regarding the types of otological diseases combined with deep learning models, there are few studies on using detection results of acoustic immittance, acoustic reflex, and pure tone hearing threshold to achieve accurate predictions, prognosis, and treatment. Additionally, while otitis media and vertigo have received significant research attention, other diseases such as otosclerosis, ear tumors, and sudden neurotropic hearing loss remain understudied. Future research on combining otological diseases with computer learning may increase the coverage of relevant studies and provide clinicians with more precise and diverse tools for diagnosis and treatment.

## Data Availability

The datasets presented in this article are not readily available because The data is not publicly available. Requests to access the datasets should be directed to moondancer122@163.com.
